# UHC in Morocco: a bottom-up estimation of public hospitals' financing size based on a costing database

**DOI:** 10.1186/s13561-024-00501-x

**Published:** 2024-04-01

**Authors:** El Houcine Akhnif, Abdelouahab Belmadani, Awad Mataria, Maryam Bigdeli

**Affiliations:** 1 World health organization/country office of Morocco, 3 Avenue S.A.R. Sidi Mohamed, Rabat, Morocco; 2 Ministry of health, Directorate of planning of financial resources, 335, Avenue Mohamed V, Rabat, Morocco; 3 World Health Organization Regional Office for the Eastern Mediterranean, Nasr City, PO Box 7608, Cairo, 11371 Egypt

**Keywords:** Hospital costing, Financial estimates, Cost containment, Health financing

## Abstract

**Background:**

Morocco is engaged in a health system reform aimed at generalizing health insurance across the whole population by 2025. This study aims to build a national database of costs at all levels of public hospitals in Morocco and craft this database as a resource for further use in a strategic purchasing system. It also aims at estimating the funding gap and the budget that should be secured for public hospitals in Morocco to fully play their roles in the current ambitious reform.

**Method:**

A costing study was implemented in 39 hospitals in 12 regions of Morocco (10 provincial hospitals, 11 regional hospitals, and 18 teaching hospitals). Using the hospital costing approach, we adapted and validated nationally our methodology to generate a database of unit costs based on data from 2019. All perspectives on cost were considered. Data collection was performed by cadres from MoH and facilitated by the WHO country office in Morocco. The production of the cost database allowed the development of a bottom-up estimation of the financing size for public health hospitals.

**Results:**

The study showed the feasibility of large-scale costing in the context of Morocco. The ownership of MoH and adherence to the process ensured the high quality of the collected data. There are many differences in unit costs for the same services moving from one hospital to another, which indicates existing inefficiencies. The database will contribute to shaping the strategic purchasing mechanism within the generalized health insurance schemes. The studied hospitals could be used as references to systematically update the billing system for health insurance.

## Key messages

- The generalization of health insurance requires more than ever a cost containment strategy.
- Strategic purchasing implementation should be based on costing approaches.
- Costing of hospital activities will help develop cost norms for more hospital efficiency.

## Background

The 2030 Sustainable Development Agenda provides a strategic framework for health systems to create interconnections with many health determinants located in other Sustainable Development Goals (SDGs). Therefore, implementing the SDGs is considered an 'indivisible whole' [1]. If most countries are committed to achieving the 2030 SDG agenda, progress has been delayed by the COVID-19 pandemic. There is a pattern of interconnectedness between SDGs that can be related to COVID-19 consequences [2]. Many researchers devoted part of their studies to the adequacy of health financing in a health system to help deal with future health crises like COVID-19 [3]. Investing in core health system functions becomes crucial, such as service delivery and health financing, with a call for increased funding for public health services and essential health functions, including infection prevention and control, surveillance, and information systems [4].

Universal Health Coverage (UHC) is confirmed globally as a solid objective for countries to achieve. It is defined as the capacity to provide all people with access to health services of sufficient quality while also ensuring that the use of these services does not expose the user to financial hardship [5]. Thus, UHC is more than ever a means to secure good health in the future. More importantly, this has increased the need to accelerate efforts to build strong and resilient health systems to achieve progress toward UHC [6]. Strengthening health systems is central to achieving UHC in many low and middle-income countries (LMICs) [7, 8] and the generalization of medical coverage schemes, especially for vulnerable and under-covered groups, including in the informal sector [9–11]. Now more than ever, funding for health systems should be made available for a quick and effective response to emergencies, requiring a supportive, flexible public financial management system [4, 6].

For any health system’s transformation, cost containment is a crucial issue, considering the scarcity of resources in general and, more specifically, in the context of LMIC. A study showed that countries that have already achieved universal access in Europe struggled with cost containment as health expenditures evolved significantly [12]. Another study compared eight countries regarding the purchasing mechanism and concluded that not all these mechanisms are strategic and influence the quality of care and performance. However, there are individual components of strategic purchasing that have an impact in terms of benefits to health systems [13]. While cost containment is essential to the sustainability of any health financing mechanism, it has to be based on sound evidence and should stem from a rationale that addresses inefficiencies in health systems while maintaining adequate access to essential services and upholding the value of equity.

A study has shown the significant impact of COVID-19 on the Moroccan economy in general, despite the tremendous efforts made by the government during the pandemic [14]. This was observed at the level of the economy’s capacity to reduce poverty and many other bottlenecks that slowed down development compared to the trend before the pandemic. A lesson learned from the COVID-19 response is to ensure that health systems adopt a broader vision that includes the system’s capacity to anticipate and respond to future threats and crises.

The COVID-19 pandemic revealed the weaknesses of the health system in Morocco, as in many other health systems in the world. However, the crisis also demonstrated considerable strengths throughout the national response [15]. Before the crisis, Morocco struggled to implement UHC actions for many years [16–19]. In the aftermath of the pandemic, a window of opportunity is now open for structural reform, not only following the global recommendation to "Build back better" but also due to a rising national political will to transform the health system. Over the last year, Morocco took historical actions, one related to generalizing health insurance for the whole population and the second to launch a structural health system reform [20]. This reform was integrated within a national vision of a New Development Model that was formulated based on a participatory approach [21].

The national health financing strategy adopted in 2021 by the Ministry of Health in Morocco highlighted the need to perform costing studies as a prerequisite for strategic purchasing [22]. A systematic review showed a positive impact of local/hospital-based HTA on hospital decisions and budgets and a positive perception from managers and clinicians [23]. Since 2002, many studies have been conducted on hospital costing, and all of them have revealed a discrepancy between the used tariffs at hospitals and the actual cost [24, 25]. One of the reasons behind this is that hospital financing was based on a social role in increasing accessibility. To provide sufficient and sustainable financing for hospitals, evidence on costing becomes more than mandatory. In this sense, it is essential to form a national team capable of carrying out studies on costs with an update over time to respond to specific needs in terms of decisions on pricing and advocacy for financing. This database must consider the variation in the supply of care between regions and provinces of the kingdom. It is, therefore, necessary to develop, in addition to a national capacity for cost analysis, a regional ability at the subnational level to organize the information needed for calculating costs.

Cost studies will also serve as a first step towards establishing a real national capacity for health technology assessment, which goes as far as conducting in-depth analyses of the costs and effectiveness of health interventions. The skills development approach of the Ministry of Health as manager of both health system reform and social protection development in the country will contribute to the overall efficiency of the UHC system.

This study aims to build a national database of costs at all levels of public hospitals in Morocco and craft this database as a resource to be further used for a strategic purchasing system. It also aims at estimating the funding gap and the budget that should be secured for public hospitals in Morocco to fully play their roles in the current ambitious reform.

## Method

We tailored our methodology to existing hospitals’ information management systems in Morocco. We used a pragmatic approach based on the most accurate strategies to estimate unit costs while considering the availability of data at the hospital level. A literature review was done to generate our conceptual framework. The review covered works and sources in different countries [26–30].

We first started by analyzing the structure of several hospitals (covering all three levels of care), and based on that, we defined the key methodological aspects. The methodology was tested through a discussion in three workshops with representatives and directors of hospitals in different regions. The development of our framework for cost calculation and analysis was inspired by the literature and adapted to the existing information system. For example, to allocate general expenditures (overheads) over direct final units, we adjusted the distribution proportions proposed by Drummond [31]. The adaptation process was done through a deep analysis of the hospital information system, and the nature of internal resource flows in hospitals.

**Fig. 1:**
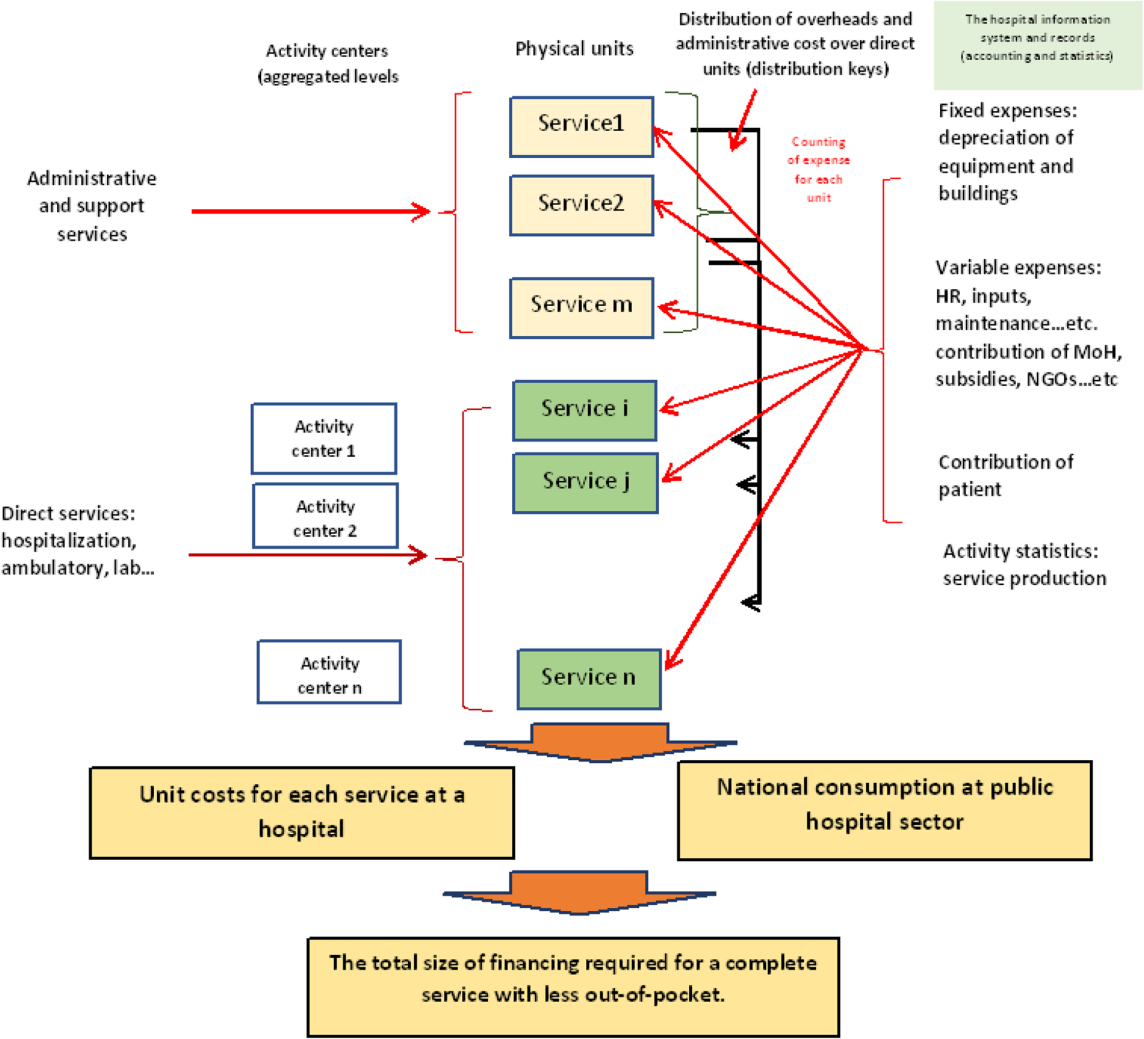
The cost calculation conceptual framework

Our framework was built on a standardized analytical structure with the same coding system. After that, we developed data collection tools to allow the counting of all resources used at all hospital units. The calculation of the capital part of the cost was done for each hospital unit. We also defined the final products of each type of unit according to the nature of the activity and unified that for all hospitals through cost centers’ coding. As part of the data collection, for each hospital unit, a quantification of the activity was included in the data collection tools. A detailed explanation of the methodological aspects is presented in the following sections: Fig. 1 presents the conceptual framework used in this study.

The "cost" was defined to include all expenses (human resources, materials, inputs…etc.) that have contributed to providing the health service at the hospital level. It also consists of all perspectives on financing (sources), including out-of-pocket expenditures.

Standardization was applied to allow comparability of unit costs between hospitals. Indeed, the change in the costing methods highly affects the results, as demonstrated in a study in Thailand [32].

### Settings

The study was developed in 2021 and was based on the latest available data in public hospitals before the COVID-19 pandemic (2019). The choice of 2019 data was based on a discussion with national officials and managers from the Ministry of Health to select data that represented a normal year of activity. Indeed, during the COVID-19 pandemic, hospital activities were reduced to care for infected patients, and the calculated cost may not represent the ordinary situation of hospital activities.

The study covered 39 hospitals at the three health system levels. The sample was composed of 18 tertiary hospitals (teaching hospitals) belonging to three regions (Fes, Oujda, and Rabat), eleven regional hospitals covering 11 regions out of 12 in Morocco, and 10 provincial hospitals in 10 regions. The sample was designed to represent all existing variations in terms of performance, size, and levels of care and was validated at the national level.

### A collaborative dynamic

The study was conducted based on a collaborative approach with the high involvement and commitment of the Ministry of Health and the expertise of the WHO country office in Morocco.

The following Table 1 presents the details of this collaborative dynamic:

**Table 1:** Details of MoH involvement in the study

• A list of motivated MoH cadres was nominated at all levels to contribute to the study.
• A central-level team of six people was in charge of supervising the data collection at the regional and provincial levels. Among them, two received training on operational methodology for cost analysis and calculation.
• For each hospital, a focal point was nominated to perform data collection at the local level under the supervision of the hospital directorate.
• Eleven regional supervisors were nominated.
• Thirty-six focal points (data collectors) received three days of training about the methodology and data collection tools, as well as the use of the study results in hospital-level decision-making. The training was delivered by WHO experts and followed by general capacity building on concepts of health system strengthening and resilience.
• Three workshops of three days each were organized in three regions (Marrakech, Fes, and Casablanca).

The study was implemented using the human resources of MoH to structure the data collection process. The complexity and nature of the data also required the commitment of regional health directors, whose country offices followed up on the process and motivated their focal points. Table1 presents how the MoH was involved in the process.

**Fig. 2:**
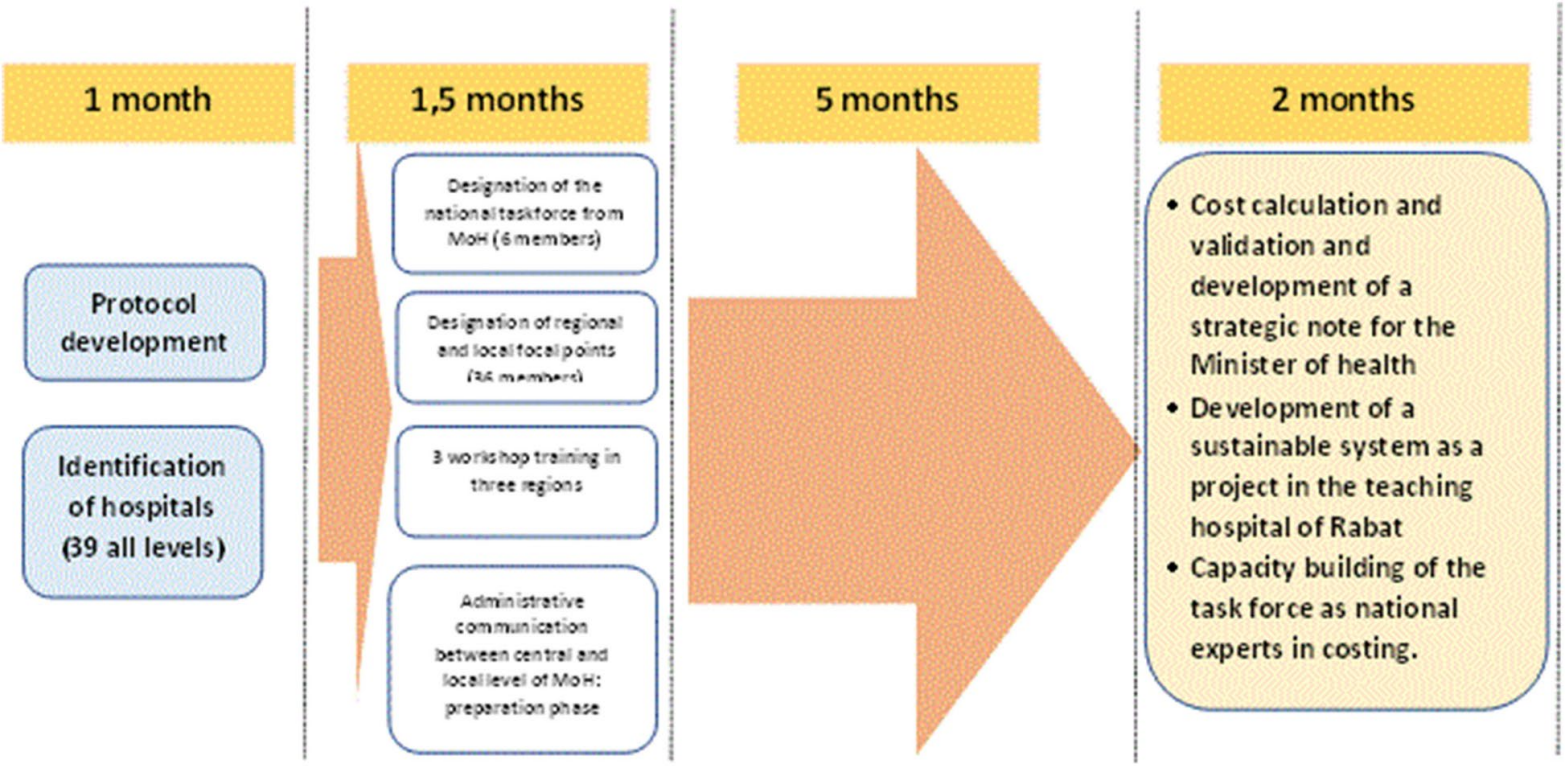
The study implementation process

Figure 2 describes the operational process from the beginning to the end.

### Key methodological aspects

#### Capital and human resources costs

Capital depreciation was calculated based on the economic depreciation method, as presented in Drummond [32]. Each hospital provided a detailed inventory of its equipment and buildings for this. At the central level of the Ministry of Health, we validated the depreciation parameters (Table 2):

**Table 2:** Capital depreciation parameters

• The life span of equipment is fixed at 10 years,
• The informatics equipment and transport at five years,
• The buildings last up to 25 years.
• The annuity factor was based on an interest rate of 5%, the most common in the costing literature.
• At the end of its lifespan, the equipment is not resold.

In all hospitals, some human resources work as volunteers or by contract. For volunteers, an average salary of a similar position level is used from the market. Also, medical staff and some nurses work in different units; their full-time salaries were split over these units through documented administrative sheets. Table 2 provides details about capital depreciation parameters.

#### Allocation of overheads and support services

To distribute overheads and support services’ costs over direct and final products, we used the list of keys (allocation proportions) validated nationally during the hospital reform since 2005. Table 3 gives an idea of the defined variables for allocation proportions.

**Table 3:** Variables used to allocate overheads to the final units and products

**General unit**	**Distribution key (rates)**
Directorate of the hospital and its offices	Equivalent full-time (EFT)
Medical services (administration)	EFT of medicals
Nursing services (administration)	EFT of nurses
Reception and information	Number of patients
Financial services	Total expenditures of each unit
Accounting service	Total expenditures of each unit
The billing service	Number of patients
Supply service and stores	The total received amount from each store
Statistic services	Number of patients
Human resources services	Equivalent full-time (EFT)
Space maintenance	M2 (square meters)
Cleaning services	M2 (square meters)
Informatic service	Number of computers
Laundry	Total cleaned weight (Kg)
Catering service	Total number of served meals
Maintenance of equipment	Number of maintenance interventions
Gardening service	M2 (square meters)
Pharmacy (HR and logistics)	Number of distributed items
Electricity bill	M2 (square meters)
Water bill	Number of patients plus HR

Using these distribution keys, we applied the cascading distribution method without iteration to ventilate all overheads and support costs over direct and final units [32].

#### Quantification of activities

To calculate unit costs, we needed to estimate the production of each hospital service. We used the units adopted in the national billing system, which consisted of hospitalization days, number of surgeries and specialized units from the nomenclature of procedures and interventions, number of tests, number of consultations in outpatient services, number of emergency visits, and number of hemodialysis sessions.

#### Standardization of aggregated levels

To allow comparison and the grouping of services by the nature of the activity, we created a coding system that all hospitals adopt. This coding system has two levels: first, the physical units according to which the hospital is organized. We created a code for activity centers that groups all similar activities, e.g., all activities related to surgery, irrespective of specialization, in one activity center called surgery hospitalization.

#### Estimation of the total size of hospitals’ financing

We used the total admissions of all hospitals in Morocco and followed the trend to calculate the total number of admissions for 2023. The estimation for 2023 was based on the analysis of last year’s evolution of the admission number, excluding the years 2020 and 2021, where, exceptionally, the pandemic reduced the use of hospital services for more focus on treating infected patients. We developed distribution keys based on our sample of 39 hospitals to split the total number among the types of activities. Based on our results, we calculated the cost of an inpatient for each aggregated activity and calculated a percentage to split the total number of admissions observed nationally. We finally added to that the cost related to ambulatory care, mainly outpatient consultations and hemodialysis sessions.

## Results

### Unit costs

The unit costs were calculated for each unit in a hospital and then aggregated at the level of the activity center that was harmonized among hospitals. For example, the surgery center might be organized differently from one hospital to another; some have general services, while others have a more precise nature of services (traumatology, ophthalmology, etc.). Table 4 presents the unit costs for each service aggregated by the level of care and by main activity centers.

**Table 4:** Unit cost for the hospital’s activity centers (aggregated by levels)

**Type of Activity**	**Level**	**Unit of measurement**	**Unit cost without depreciation**	**Unit cost with the depreciation**
**Surgery hospitalization**	Provincial hospital	hospitalization day	$58,49	$74,93
**surgery hospitalization**	Regional hospital	hospitalization day	$73,43	$83,15
**surgery hospitalization**	University Hospital	hospitalization day	$79,41	$88,03
**Medicine hospitalization**	Provincial hospital	hospitalization day	$61,14	$68,41
**Medicine hospitalization**	Regional hospital	hospitalization day	$74,69	$82,06
**Medicine hospitalization**	University Hospital	hospitalization day	$67,64	$74,62
**Maternity**	Provincial hospital	hospitalization day	$66,48	$73,25
**Maternity**	Regional hospital	hospitalization day	$81,41	$91,63
**Maternity**	University hospital	hospitalization day	$74,33	$84,69
**Neonatology hospitalization**	Provincial hospital	hospitalization day	$38,69	$42,50
**Neonatology hospitalization**	Regional hospital	hospitalization day	$81,13	$93,47
**Neonatology hospitalization**	University hospital	hospitalization day	$127,26	$133,29
**Pediatrics hospitalization**	Provincial hospital	hospitalization day	$38,02	$43,47
**Pediatrics hospitalization**	Regional hospital	hospitalization day	$91,08	$102,03
**Pediatrics hospitalization**	University hospital	hospitalization day	$96,71	$103,43
**Psychiatric hospitalization**	Provincial hospital	hospitalization day	$27,44	$29,96
**Psychiatric hospitalization**	Regional hospital	hospitalization day	$25,99	$30,13
**Psychiatric hospitalization**	University hospital	hospitalization day	$59,11	$62,68
**ICU**	Provincial hospital	hospitalization day	$149,75	$183,06
**ICU**	Regional hospital	hospitalization day	$319,67	$465,40
**ICU**	University hospital	hospitalization day	$394,83	$453,94
**Emergency**	Provincial hospital	Consultation	$14,15	$15,53
**Emergency**	Regional hospital	Consultation	$13,86	$16,59
**Emergency**	University hospital	Consultation	$64,92	$69,86
**Outpatient consultation**	Provincial hospital	Consultation	$29,27	$30,72
**Outpatient consultation**	Regional hospital	Consultation	$28,72	$29,72
**Outpatient consultation**	University hospital	Consultation	$39,69	$42,71
**Radiology**	Provincial hospital	Unit Z	$1,00	$1,49
**Radiology**	Regional hospital	Unit Z	$0,57	$0,92
**Radiology**	University hospital	Unit Z	$0,44	$0,52
**Laboratory**	Provincial hospital	Unit B	$0,13	$0,17
**Laboratory**	Regional hospital	Unit B	$0,08	$0,11
**Laboratory**	University hospital	Unit B	$0,07	$0,07
**Operating theater**	Provincial hospital	Unit K	$6,75	$7,57
**Operating theater**	Regional hospital	Unit K	$5,76	$8,20
**Operating theater**	University hospital	Unit K	$2,59	$3,07
**Hemodialysis**	Provincial hospital	session	$56,21	$64,92
**Hemodialysis**	Regional hospital	session	$62,07	$73,31
**Hemodialysis**	University hospital	session	$182,68	$186,15

The hospital day for surgery ranges from 75 USD for a provincial hospital to 88 USD for a teaching hospital. For medical hospitalization, a one-day stay costs 68, to 75 USD, respectively, for local, and teaching hospitals. As for the maternity ward, a day's stay costs from 73 to 85 USD, respectively, from provincial to teaching hospitals. The unit cost for a one-day stay for the neonatology activity ranges from 42 to 133 USD. One-day stays in the pediatric activity center, range from 43 to 102 USD. The psychiatric activity center has unit costs ranging from 29 to 62 USD. The unit cost of a one-day stay at the ICU ranges from 183 to 453 USD. One consultation's unit cost varies from 15 to 69 USD. Outpatient consultations cost 31 TO 42 USD. One radiology unit (Z according to the nomenclature of interventions) costs from 1,5 TO 0,52 USD, showing an increase in the unit cost as we move to higher levels of care correlated to the size of activity and the efficiency in using the capital cost. Activity (B) costs from 0.17 to 0.07 USD in the laboratory unit, with the same trends as the radiology activity. Operating theater activity for the surgery unit (K) costs 7.57 USD to 3.07 USD. One session of hemodialysis costs 65 to 186 USD.

### Detailed unit costs for tracer activities

**Fig. 3:**
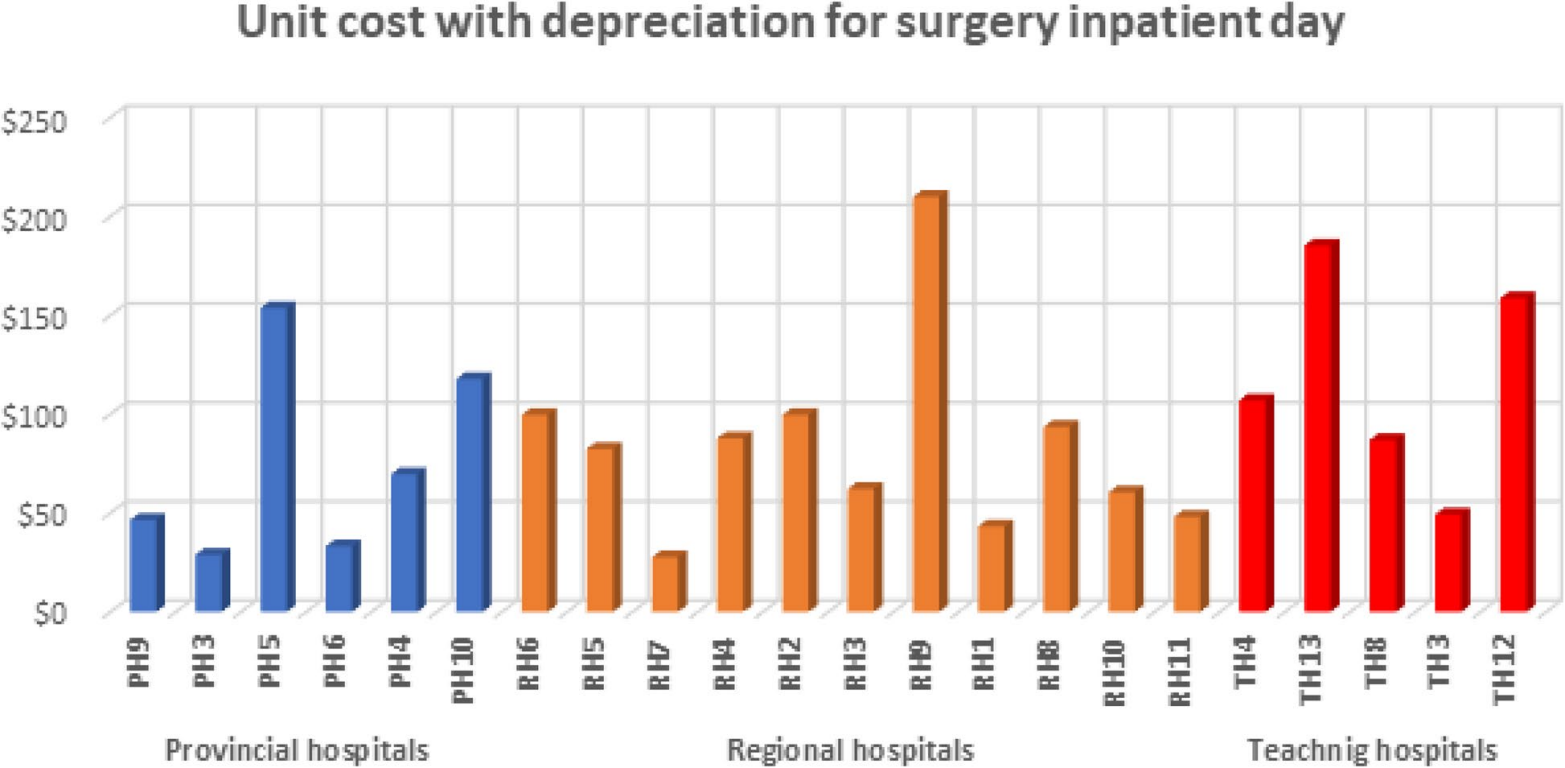
The variation of the unit cost for an inpatient day in surgery services by hospital

**Fig. 4:**
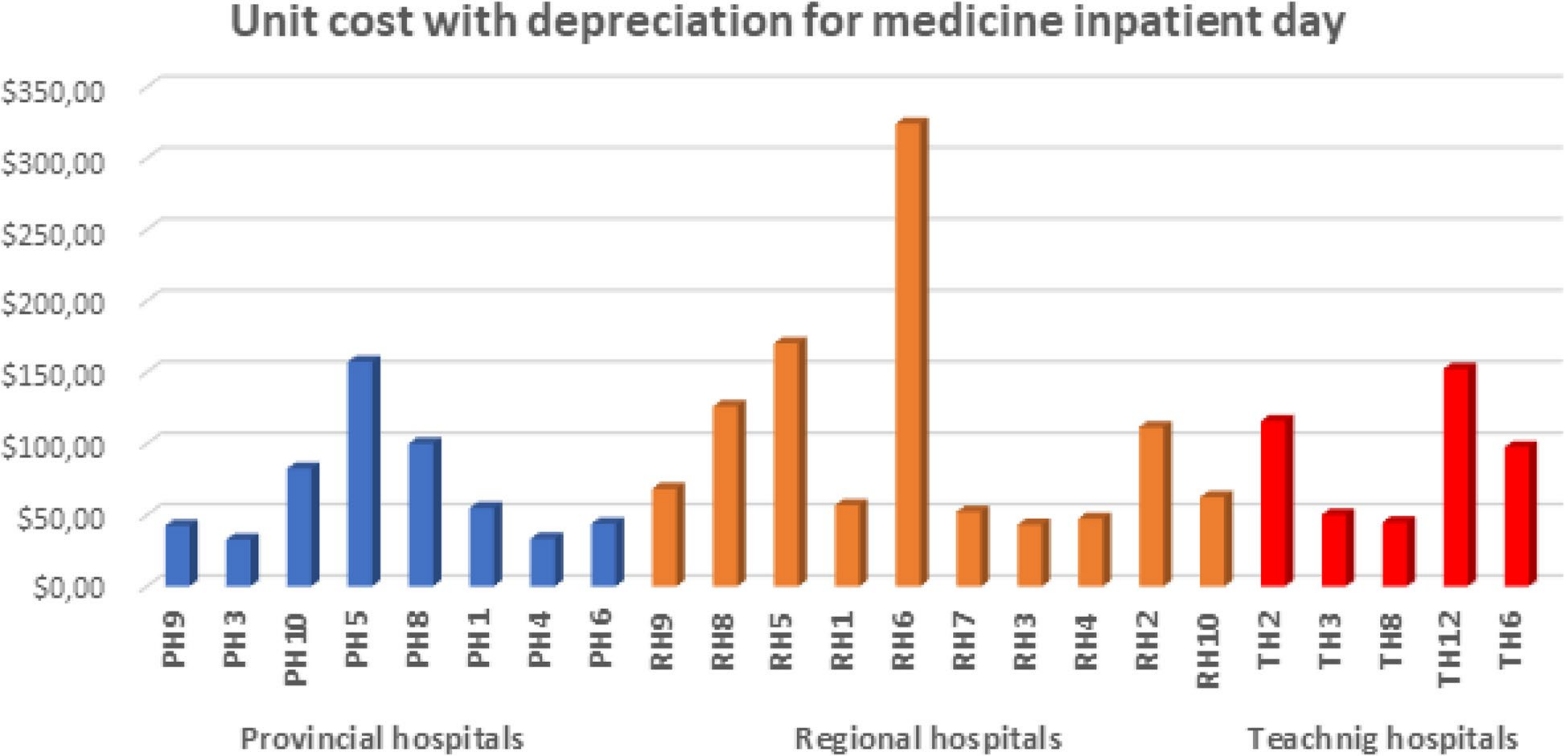
The variation of the unit cost for an inpatient day in medicine services by hospital

**Fig. 5:**
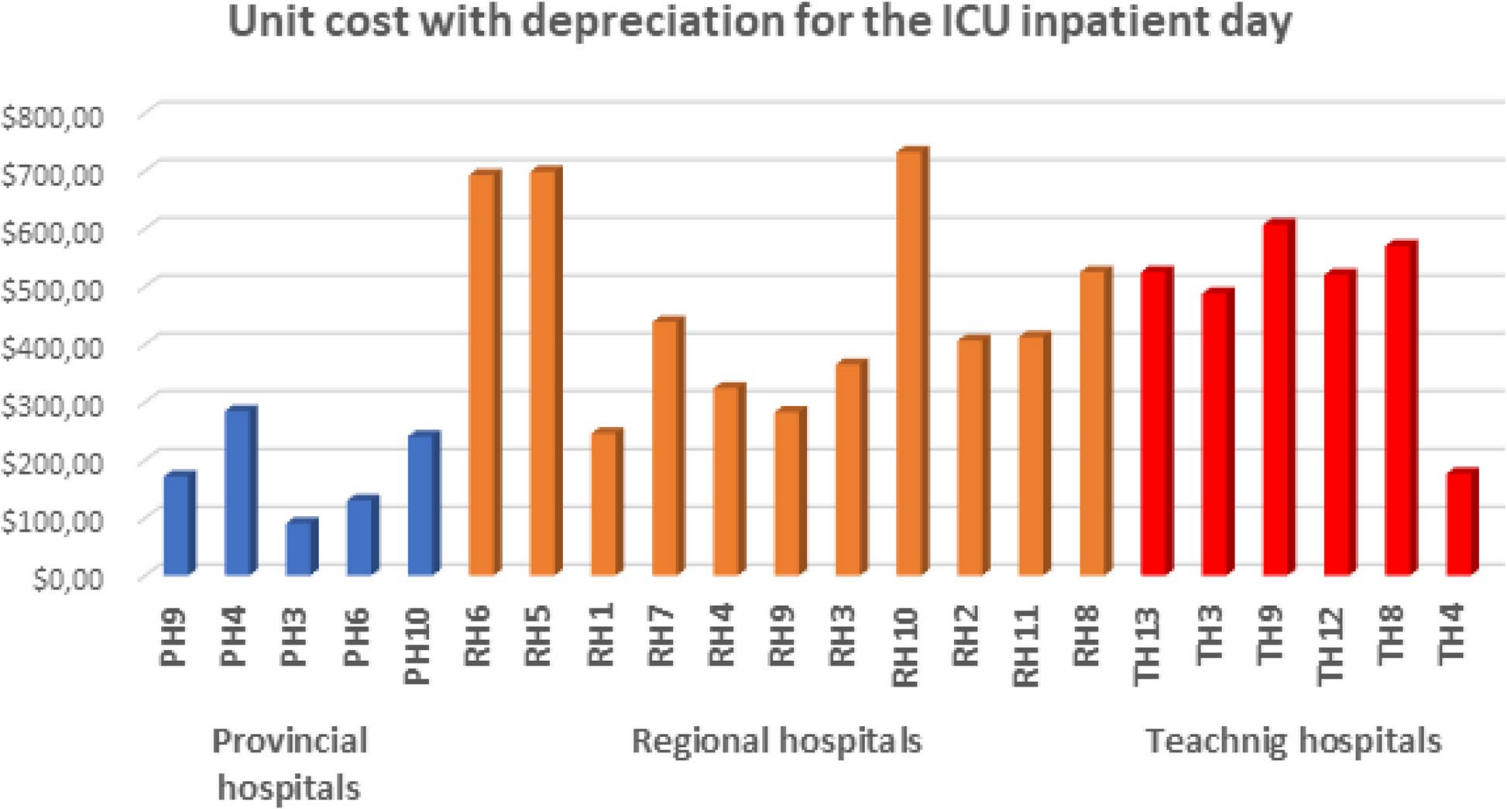
The variation of the unit cost for an inpatient day in ICU services by hospital

**Fig. 6:**
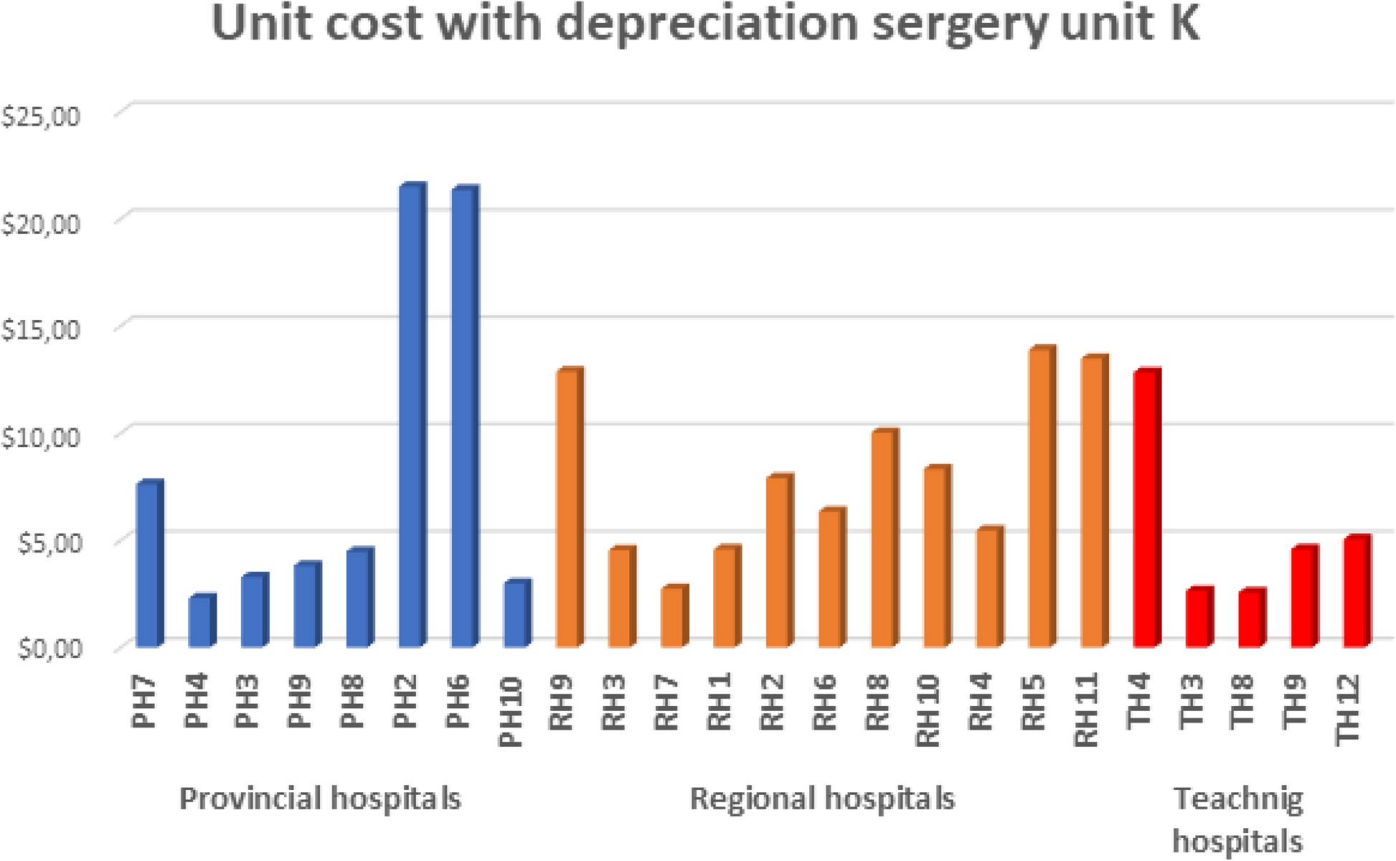
The variation of the unit cost for a unit K of surgery by the hospital

The following figures (Figs. 3, 4, 5 and 6) provide the detailed unit cost of hospitalization activities for each hospital and tracer activity.

Figure 3 shows that for provincial hospitals, the unit cost of an inpatient day in surgery varies from 29 to 154 USD, with an important variation among hospitals at the regional level. The unit cost for the inpatient day varies from 28 to 210 USD. As for the tertiary level, the unit cost for an inpatient day of surgery ranges from 49 to 185 USD. The number of admissions is crucial in determining the unit cost, as capital expenditures are maximized according to the production size.

According to Fig. 4 for the provincial hospitals regarding the activity of medicine, the unit cost ranges from 32 to 157 USD for the inpatient day, with a significant variation among hospitals. As for regional hospitals, the variation is also significant, and the unit cost varies from 43 to 324 USD. The unit cost for teaching hospitals ranges from 45 to 152 USD. The variation in unit cost for the same nature of the activity is quite important and indicates the existence of room for improving efficiency.

Figure 5 shows that the variation of the unit cost for one inpatient day in the ICU is presented in Fig. 5. For provincial hospitals, the unit cost of an inpatient day stay varies from 90 to 284 USD. For regional hospitals, the unit cost of a day's stay ranges from 246 to 732 USD, significantly varying among hospitals. As for the teaching hospitals, the unit cost ranges from 175 USD (for an ICU for children) to 606 USD. For the teaching hospitals, the difference among hospitals is insignificant.

Figure 6 presents the unit cost for one unit K of surgery. The K unit is a virtual unit defined by Morocco's general Nomenclature of health procedures. The activity of the operating theater is measured by the number of K units, which is more accurate than using the number of interventions, where complexity differences will be masked. For provincial hospitals, the unit cost of one unit K ranges from 2.31 to 21.5 USD. For the secondary level, the unit cost for one surgery unit K varies from 2.75 to 14 USD. At the tertiary level, one unit K's cost ranges from 2.57 to 13 USD. We observe a considerable variation in the unit cost within the same level of care but also moving from one level to another.

### Proportion pharmaceutical products in the unit cost

As pharmaceutical products are important in the process of care at the hospital level, we examined their proportion in the total unit cost for each type of service. Table 5 provides the percentage of pharmaceuticals in the total unit cost.

**Table 5:** The proportion of pharmaceutical products in the unit cost

**Service**	**Proportion of pharmaceutical products in the unit cost**
Medicine	31%
surgery	15%
Maternity and obstetrics	13%
pediatric	33%
ICU	26%
Neonatology	20%
Psychiatry	3%
Outpatient consultation	6%
Hemodialysis	22%
Oncology	56%
Emergency consultation	20%
Radiology	19%
Laboratory	60%
operation theater	20%

The proportion of pharmaceutical products ranges from 3% in the psychiatric ward and 6% for outpatient consultations where human resources cost is important, and 60% for oncology services where the drug costs more than the rest of the cost components.

### Estimation of the financing size for the tertiary level (teaching hospitals)

Based on data collected from MoH databases, we estimated the trend in the number of admissions at each level. Using the 39 hospitals, we identified the frequencies for each type of service for one admission (patient), and we established an aggregated cost per admission through the costing database and using the coding system. The total number of admissions for 2023 was estimated at around 306.706; using the frequencies, we ventilate this number over the categories of main health activities as mentioned in Table 5. Finally, the total cost was calculated as the size of the financing for the tertiary level.

The total financing size is estimated at 814.273.550 USD according to this bottom-up approach for the tertiary level of care. This financing estimation includes all inpatient services and considers laboratory and radiology services.

### Estimate of the size of regional hospitals

Table 6 presents the estimation of the financing size for regional hospitals based on the same approach as explained before.

**Table 6:** Estimation of the total size of the financing for teaching hospitals

**Activity type**	**Unit cost**	**Frequency**	**Total number of inpatients (teaching hospitals)**	**inpatient by nature of the activity**	**Total Cost**
Surgery (hospitalization and interventions)	$2.690,09	23,19%	**306.706**	71127	$191.340.034,88
Maternity	$195,19	36,65%		112405	$21.940.268,00
medical hospitalization	$1.978,48	20,67%		63403	$125.442.839,96
Oncology	$10.681,18	1,10%		3367	$35.963.978,95
Pediatric	$873,08	9,45%		28994	$25.314.065,00
ICU	$2.113,95	8,94%		27407	$57.938.942,66
**Activity type**	**Unit cost**	**Average units consumed by inpatient**		**Total Cost**	
laboratory	$0,08	2529		$59.185.229,65	
Radiology	$0,52	165		$26.527.775,70	
**Total Size of financing for teaching hospitals**	**$814.273.550,81**				

The total amount of financing for regional hospitals is estimated at 97.992.516 USD.

### Estimate for the financing size of provincial hospitals

Table 7 presents the cost and estimation of the total financing necessary for the functioning of provincial hospitals.

**Table 7:** Estimation of the total size of the financing for regional hospitals

**Activity type**	**Unit cost**	**Frequency**	**Total number of inpatients (Regional hospitals)**	**inpatient by nature of the activity**	**Total Cost**
Surgery (hospitalization and interventions)	$626,52	23,19%	**217 419**	71127	$35.837.714,14
Maternity	$108,86	36,65%		112405	$8.987.792,61
medical hospitalization	$493,61	20,67%		63403	$16.970.667,65
Pediatric	$157,45	9,45%		28994	$5.861.897,66
ICU	$1.323,68	8,94%		27407	$8.003.759,01
**Activity type**	**Unit cost**	**Average units consumed by each inpatient**		**Total Cost**	
laboratory	$0,07	2529		$10.073.955,05	
Radiology	$0,86	165		$12.256.730,31	
**Total Size of financing for regional hospitals**	**$97.992.516,80**				

### Estimate for outpatient consultations’ cost

We calculated an aggregated cost for all hospitals to estimate the total cost necessary for producing outpatient consultations. We generated the trend regarding the total number of consultations based on MoH records and databases (Tables 8 and 9).

**Table 8:** Estimation of the total size of the financing for regional hospitals

**Activity type**	**Unit cost**	**Frequency**	**Total number of inpatients (provincial hospitals)**	**inpatient by nature of the activity**	**Total Cost**
Surgery (hospitalization and interventions)	$577,53	23,19%	**665517**	71128	$64.505.236,12
Maternity	$109,41	36,65%		112405	$28.814.455,06
medical hospitalization	$284,16	20,67%		63404	$42.810.286,92
Pediatric	$115,98	9,45%		28994	$13.024.269,03
ICU	$335,12	8,94%		27408	$9.220.937,35
**Activity type**	**Unit cost**	**Average units consumed by each inpatient**		**Total Cost**	
Laboratory	$0,15	2529		$23.471.282,34	
Radiology	$0,73	165		$18.104.484,09	
**Total Size of financing for provincial hospitals**	**$199.950.950,91**

**Table 9:** Estimation of the total size of the financing for outpatient consultations

**Activity type**	**Unit cost**	**Total number of outpatients**	**Total Cost**
**Outpatient consultations in all hospitals (all levels)**	$21,05	**3.855.107**	$81.160.148,25

The total financing necessary for producing outpatient consultations in all hospitals for 2023 is estimated at 81.160.148 USD.

### The total size of public hospitals’ financing in 2023

By adding the total cost of all levels of care, the total financing required for all hospitals in Morocco in 2023 is $1.193.377.166,77**.** This financing size considers an average of consumption translated into the unit cost for each type of admission. By working on efficiency, the financing needs could be reduced, or alternatively, the funding could be provided in full and used to improve the quality of care.

## Discussion

Our first objective was to demonstrate the feasibility of a large costing study with an impact on estimating the size of required financing in hospitals in the Moroccan context. Indeed, the information system has many areas for improvement, making complex costing studies challenging. The approach we adopted by engaging local and central actors in the process created ownership with an intrinsic motivation to acquire costing expertise. Data availability and cost capacity challenges are not unique to the Moroccan system. The French reform identified several hospitals for which the billing system was initiated in view of enabling accurate costing exercises to accompany the medicalization of the information system program [33, 34]. Training focal points on the details of the costing methodology contributed to improving the accuracy of the collected data and the validation of the study's final results. It is possible to conduct a costing exercise using available hospital data, although it is likely that the data quality will need to be improved over time. For example

We observed differences in the costs of the same activities moving from one hospital to another within the same level of care. These differences are normal and depend on factors such as the size of the facility, its range of activity, the quality of care, the differences in the characteristics of the patients, and production factors [35]. The study also revealed differences between levels of care. Again, it is expected to observe an increase in unit costs as we move from provincial and regional to teaching hospitals. However, the study also revealed some surprising results, including higher unit costs for the same service at the provincial hospital than in teaching hospitals. Our analyses showed that in some remote and nonattractive hospitals, the utilization of services is low, which reduces the overall productivity of the hospital and increases the unit cost. This indicates problems related to efficiency that were not identified before this study. The study results can be used to point out inefficiencies in selected hospitals and monitor them by repeating the exercise regularly. Only in this way does the study support organizational learning for hospitals to address these efficiencies. Also, for health insurance, having an idea about the unit cost variation may provide more insights into developing an efficient billing system and introducing a strategic purchasing system. Indeed, Morocco is engaged in a radical health system reform that will certainly require a considerable mobilization of resources. Efficiency at all levels will reduce the burden on public finances and mobilize more resources. Pharmaceutical products are important in the care process at the hospital level and determine in some cases the total cost. The efficiency will impose some strategies as to the adoption of therapeutic protocols that are efficient to reduce the cost and cover more patients by the already insufficient resources. During 2022 the health insurance agency in Morocco designed several protocols that will condition the reimbursement process for more care effectiveness and efficiency. Introducing the Health Technology Assessment as a tool will help bring an economic vision to the clinical decisions concerning the use of pharmaceutical products in hospitals.

We observed that some services cost less at teaching hospitals because of their high productivity. When unit costs are compared to the rates used in the hospital billing system, we notice that the actual rates would not allow the hospital to cover its costs and thus continue to serve its population in the coming years. It's important to mention that hospital rates were defined based on a social approach to allow for increasing utilization and reducing financial barriers. The generalization of health insurance created a separation between the financing and service delivery functions, which will allow the introduction of more intelligent purchasing arrangements that are strategic and conducive to efficiency improvement. Costing studies will be crucial in unveiling the situation and identifying efficiency problems. Therefore, our study findings can support a specific purchasing mechanism in Moroccan hospital systems; for example, using the DRG system as applied in several other countries will improve hospital efficiency [36]. The detailed database will be useful in costing the DRGs and building more efficient strategic purchasing. Best practices in health systems highly recommend strategic purchasing. Increasing productivity at all levels of care, combined with a costing system, will support hospitals in achieving the most cost-effective organizational modes. The hospital's efficiency will contribute to the overall health system's efficiency and increase the fiscal space for health through efficiency [37].

Detailed costing allowed us to estimate the total necessary cost for each level of care, which will provide a vision for decision-makers and the health insurance fund managers to analyze the feasibility of increasing or decreasing the benefits package. By examining cost details, we conclude that human resource productivity is crucial to reducing costs.

The costing database allows for a discussion on prioritizing the benefits package interventions based on the financial feasibility. Indeed, the cost of each intervention to be included in the benefits package will require a deep analysis of the economic impact of providing this service to the whole population. The bottom-up approach we developed throughout this study will facilitate the analysis of all interventions and judge whether the introduction of a service will impact the overall UHC financing system. The estimation of the financing size could also be used to advocate for additional resources for the health system or the health insurance funds.

## Conclusion

Morocco is preparing performance-based financing for hospitals, which could boost productivity and increase efficiency. The costing system supported by this study can be used to observe the impact of financial incentives on reducing unit costs and improving the overall efficiency of the health system. The current reform of the health system and the generalization of health insurance will require mechanisms and tools to estimate the actual financial needs of hospitals. To preserve the continuity of public service, a steady increase in fiscal space will allow for the consideration of the new functions attributed to hospitals due to the reform. We can increase the fiscal space by increasing mobilized resources for health (for example, increasing rates) or increasing internal efficiency [38]. In Morocco, the new configuration of hospitals will have to integrate monitoring of costs and efficiency into their new management system. Our finding also demonstrated the possibility of estimating the overall financing size the health system should provide to minimally ensure the existing level, quantity, and quality of services to cover the insured population. The financing size will allow for testing the government's effort to generalize health insurance by comparing it with the actual mobilized resources. The study's findings will provide a systematic anticipatory tool to alert policymakers before deficits occur. This study was the first step in generating an initial database to provide decision-makers visibility. The replication of this study in the same hospitals in the future will allow the development of norms about the cost at different levels and create a reference for accountability based on efficiency. This will also generate organizational learning between hospitals by examining strategies and intelligent working teams to achieve higher levels of efficiency. We succeeded in training national experts on cost at all levels used in efficiency analysis; the hospital system will increase budgetary space considerably.

## Data Availability

The database about costing is available at the Ministry of Health and will be available upon request to the first author.

## Abbreviations

SDG: Sustainable development goals
LMIC: Low and middle-income countries
UHC: Universal health coverage
HTA: Health technology assessment
MoH: Ministry of Health
PH: Provincial hospital
RH: Regional hospital
TH: Teaching hospital
